# Supplementary material for: Association between Secondhand Smoke Exposure and Hypertension in 106,268 Korean Self-Reported Never-Smokers Verified by Cotinine
Source: J Clin Med. 2019 Aug 16;8(8):1238. doi: 10.3390/jcm8081238 (PMC6723357; doi:10.3390/jcm8081238)
Supplement: Supplementary file 1 [file jcm-08-01238-s001.pdf]

Supplementary Materials

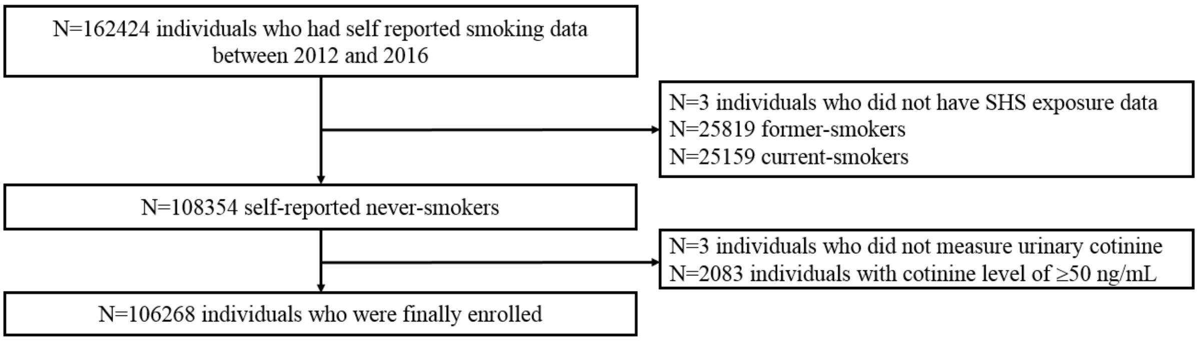

Figure S1. Flow chart for sample selection criteria. SHS, secondhand smoke.

Table S1. Characteristics of never-smokers with and without hypertension.

	**Hypertension (-)** *n* **= 100,322**	**Hypertension (+)** *n* **= 5,755**	*p* **value**
Age, years	34.0 ± 6.6	41.1 ± 11.8	<0.001
Sex (males), *n* (%)	29,127/100,322 (29.0)	3424/5755 (59.5)	<0.001
Body mass index, kg/m^2	22.2 ± 3.2	25.6 ± 4.0	<0.001
Waist circumference, cm	77.6 ± 9.2	87.1 ± 10.4	<0.001
Systolic blood pressure, mmHg	103.1 ± 10.7	125.5 ± 15.0	<0.001
Diastolic blood pressure, mmHg	65.7 ± 8.0	81.9 ± 12.0	<0.001
Serum creatinine, umol/L	67.7 ± 15.3	77.9 ± 27.1	<0.001
Uric acid, umol/L	287.1 ± 79.7	343.0 ± 91.3	<0.001
Total cholesterol, mmol/L	4.82 ± 0.83	5.14 ± 0.94	<0.001
Triglyceride, mmol/L	0.82 (0.62, 1.15)	1.20 [0.85, 1.75)	<0.001
HDL cholesterol, mmol/L	1.63 ± 0.40	1.43 ± 0.38	<0.001
LDL cholesterol, mmol/L	2.92 ± 0.78	3.30 ± 0.87	<0.001
Glucose, mmol/L	5.05 ± 0.56	5.56 ± 1.16	<0.001
hsCRP, mg/L	0.4 (0.2, 0.7)	0.6 (0.3, 1.3)	<0.001
Vigorous exercise, *n* (%)			<0.001
none/week	65,852/99,279 (66.3)	3300/5647 (58.4)	
< 3 times/week	22,168/99,279 (22.3)	1508/5647 (26.7)	
≥ 3 times/week	11,269/99,279 (11.3)	839/5647 (14.9)	
Daily alcohol consumption, gram	4.0 (1.0, 10.0)	5.0 (1.0, 14.3)	<0.001
Alcohol consumption, *n* (%)			<0.001
none/week	14,795/96481 (15.3)	887/5,442 (16.3)	
1–2 times/week	74,428/96,481 (77.1)	3898/5442 (71.6)	
3–4 times/week	6248/96,481 (6.5)	567/5442 (10.4)	
≥5 times/week	1010/96,481 (1.0)	90/5442 (1.7)	
Daily times of SHS exposure, *n* (%)			<0.001
None	78,881/91,488 (86.2)	4222/5128 (82.3)	
<1 hour/day	8629/91,488 (9.4)	582/5128 (11.3)	
≥1 hour/day	3978/91,488 (4.3)	324/5128 (6.3)	
Frequency of SHS exposure, *n* (%)			<0.001
None	78,819/100,017 (78.8)	4213/5720 (73.7)	
<3 times/week	13,947/100,017 (13.9)	944/5720 (16.5)	
≥3 times/week	7251/100,017 (7.2)	563/5720 (9.8)	
Duration of SHS exposure, *n* (%)			<0.001
None	78,596/93,291 (84.2)	4190/5243 (79.9)	

< 10 years	6044/93,291 (6.5)	365/5243 (7.0)	
≥ 10 years	8651/93,291 (9.3)	688/5243 (13.1)	
Home SHS before age 20, *n* (%)	48,626/100,322 (48.5)	2910/5755 (50.6)	0.002
Home SHS after age 20, *n* (%)	27,987/99,694 (28.1)	1706/5723 (29.8)	0.005
SHS exposure, *n* (%)	21,441/100,322 (21.4)	1533/5755 (26.6)	<0.001
Ani-hypertensive medication(s), *n* (%)	0/100,322 (0)	2060/5755 (35.8)	<0.001

Data are shown as mean ± standard deviation, median (interquartile) or percentage. Triglyceride, hsCRP, and daily alcohol amount were log-transformed for this analysis. *p* values were based on Student's t-test or Chi-square test. Secondhand smoke exposure was defined as current exposure to passive smoking indoors at home or the workplace. SHS, secondhand smoke; *n*, number; HDL, high-density lipoprotein; LDL, low-density lipoprotein; hsCRP, high-sensitivity C-reactive protein.

Table S2. Multivariate logistic regression analyses for the association of hypertension using 2017 ACC/AHA high blood pressure clinical practice guideline [a] with secondhand smoke exposure in never-smokers.

		Odds ratios (95% Confidence interval)	
	Cases of hypertension [b]	Age-adjusted	Multivariate
Overall [c]			
SHS exposure (-)	9,083/83,103 (10.9)	1	1
SHS exposure (+) [d]	3153/22,974 (13.7)	1.17 (1.11, 1.22)	1.09 (1.03, 1.14)
Males			
SHS exposure (-)	5,518/24,019 (23.0)	1	1
SHS exposure (+) [d]	2118/8,532 (24.8)	1.13 (1.06, 1.20)	1.06 (1.00, 1.13)
Females			
SHS exposure (-)	3,565/59,084 (6.0)	1	1
SHS exposure (+) [d]	1035/14,442 (7.2)	1.21 (1.12, 1.31)	1.12 (1.02, 1.21)

[a] Hypertension was defined as systolic/diastolic blood pressure ≥ 130/80 mmHg using 2017 ACC/AHA high blood pressure clinical practice guideline [18]. [b] Values are expressed as n/N (%), where n is number of cases with a given variable and N is total number considered for that variable. [c] Models adjusted for sex in overall population. [d] Reference group is group without SHS exposure. Multivariate model was adjusted for age, waist circumference, body mass index, frequency of alcohol drinking, frequency of vigorous exercise, glucose, creatinine, uric acid, total cholesterol, high-density lipoprotein cholesterol, low-density lipoprotein cholesterol, triglyceride, and high-sensitivity C-reactive protein. SHS, secondhand smoke.
